# Nano-Bio Interaction between Blood Plasma Proteins and Water-Soluble Silicon Quantum Dots with Enabled Cellular Uptake and Minimal Cytotoxicity

**DOI:** 10.3390/nano10112250

**Published:** 2020-11-13

**Authors:** Shanmugavel Chinnathambi, Nobutaka Hanagata, Tomohiko Yamazaki, Naoto Shirahata

**Affiliations:** 1 International Center for Young Scientists, National Institute for Materials Science (NIMS), 1-2-1 Sengen, Tsukuba, Ibaraki 305-0047, Japan; 2 Institute for Integrated Cell-Material Sciences (ICeMS), Institute for Advanced Study, Kyoto University, Kyoto 606 8501, Japan; 3 Nanotechnology Innovation Station, NIMS, 1-2-1 Sengen, Tsukuba, Ibaraki 305-0047, Japan; hanagata.nobutaka@nims.go.jp; 4 Graduate School of Chemical Sciences and Engineering, Hokkaido University, Sapporo 060-0814, Japan; yamazaki.tomohiko@nims.go.jp; 5 Research Center for Functional Materials (RCFM), NIMS, 1-2-1, Sengen, Tsukuba 305-0047, Japan; 6 International Center for Materials Nanoarchitectonics (WPI-MANA), NIMS, Namiki, Tsukuba 305-0044, Japan; 7 Department of Physics, Chuo University, 1-13-27 Kasuga, Bunkyo, Tokyo 112-8551, Japan

**Keywords:** silicon, quantum dots, plasma proteins, conformational changes, cell imaging

## Abstract

A better understanding of the compatibility of water-soluble semiconductor quantum dots (QDs) upon contact with the bloodstream is important for biological applications, including biomarkers working in the first therapeutic spectral window for deep tissue imaging. Herein, we investigated the conformational changes of blood plasma proteins during the interaction with near-infrared light-emitting nanoparticles, consisting of Pluronic F127 shells and cores comprised of assembled silicon QDs terminated with decane monolayers. Albumin and transferrin have high quenching constants and form a hard protein corona on the nanoparticle. In contrast, fibrinogen has low quenching constants and forms a soft protein corona. A circular dichroism (CD) spectrometric study investigates changes in the protein’s secondary and tertiary structures with incremental changes in the nanoparticle concentrations. As expected, the addition of nanoparticles causes the denaturation of the plasma proteins. However, it is noteworthy that the conformational recovery phenomena are observed for fibrinogen and transferrin, suggesting that the nanoparticle does not influence the ordered structure of proteins in the bloodstream. In addition, we observed enabled cellular uptake (NIH3T3 Fibroblasts) and minimal cytotoxicity using different cell lines (HeLa, A549, and NIH3T3). This study offers a basis to design QDs without altering the biomacromolecule’s original conformation with enabled cellular uptake with minimal cytotoxicity.

## 1. Introduction

Near-infrared (NIR) fluorescence light, which is broadly utilized for monitoring and imaging in minimally invasive medicine, provides researchers and surgeons highly specific images of target tissues in the living body [1,2,3,4,5]. Although whole-body preoperative imaging of single-photon emission computed tomography (SPECT) and positron emission tomography (PET) has diagnostic utility, such imaging lacks the appropriate resolution, sensitivity, efficacy, and targeted NIR fluorophores to simultaneously facilitate real-time delineation of the target tissue while preserving vital tissues [6]. The optical communication in the first biological window (λ = 700–950 nm) is useful in terms of minimal autofluorescence, possible depth imaging (~1.5 mm), high transparency against tissues, the low absorption coefficient of water (700–900 nm), and low light scattering [7].

Organic dyes have widely been utilized as fluorescence biomarkers. However, most are not NIR-light emitters but visible-light ones. Additionally, organic dyes exhibit a fast photobleaching character, preventing long-term fluorescence tissue monitoring upon excitation [8]. As an alternative, researchers choose semiconductor quantum dots (QDs) because of their high quantum yield and low photobleaching characters. Previously studied QDs that exhibit a molecular interaction with proteins consist of toxic heavy metal ions, such as cadmium, lead, selenium, or mercury [9,10,11].

Silicon (Si) is widely used in the device industry because it is abundant, inert, environmentally friendly, and non-toxic [12,13,14,15]. Therefore, SiQDs are a developing class of quantum materials for biomedical applications due to their superior optical properties, such as the tunability of the photoluminescence (PL) peak wavelength in the first biological window, a long PL decay time on the µsec scale to avoid overlapping imaging of autofluorescence, a high quantum yield (QY) of PL, and a high resistance to the light irradiation and changes in pH.7 [16,17,18,19]. Additionally, SiQDs might be excreted in the urine [20]. In the current work, Pluronic F127 (Food and Drug Administration (FDA) -approved amphiphile) was used as a coating agent to prepare high water dispersibility and biocompatible SiQDs. Besides, the poly (propylene oxide) block of F127 helps to the high affinity with hydrophobic SiQDs shell.

Human blood plasma contains thousands of proteins with specific functionalities. According to the human proteome organization, 89% of proteins detected from 21,842 plasma proteins have been identified. However, less than 10% have been measured quantitatively [21]. Blood contains high molecular weight proteins, such as albumin and globulin, which hamper the detection of low molecular weight proteins.

Albumin, fibrinogen, and transferrin are essential proteins found in the blood. Albumin is the most abundant protein (55%) in the blood plasma. It involves transporting and delivering fatty acids, steroids, nutrients, and several therapeutic drugs. Albumin also controls the pH and colloid osmotic pressure of blood [22,23,24]. Fibrinogen is a large cylindrical glycoprotein (7%) with a molecular weight of 340 kDa. It is an essential component for blood clots’ formation as thrombin converts it to fibrin [25]. Transferrins (<1%) are iron-binding blood plasma glycoproteins that control the level of free iron in biological fluids [26].

Three major plasma proteins mentioned above were chosen to investigate their morphological changes when they meet with nanoparticles of SiQDs. Once nanoparticles enter the bloodstream, they interact with biomolecules to form a bio-corona, such as protein corona, on their surfaces [27,28,29,30,31,32,33,34,35]. Revealing the underlying mechanism of the interaction with plasma proteins plays a significant role in nanoparticles in developing medical diagnosis and drug delivery applications [36]. However, a detailed study on the plasma proteins interacting with NIR-SiQDs is lacking. Here, we demonstrate the synthesis of water-borne NIR-SiQDs and their interactions with blood plasma proteins, specifically albumin, fibrinogen, and transferrin. Fluorescence spectroscopic analysis is used due to its sensitivity toward the microenvironment around aromatic amino acids (tryptophan-trp, tyrosine-tyr, and phenylalanine-phe) present in proteins. The secondary structure changes of the plasma proteins are measured using circular dichroism (CD) spectroscopy and analyzed by BESTSEL online software. In addition to the above experiments, we demonstrated the nanomaterials’ cell viability test using three different types of cells (HeLa, A549 (cancer), and NIH3T3 (fibroblast)) and cellular uptake (NIH3T3) to check its compatibility.

## 2. Materials and Methods

### 2.1. Materials

Triethoxysilane (TES) was purchased from Tokyo Chemical Industry and used as received. 1-Decene and proteins were purchased from Sigma Aldrich (St.Louis, MO, USA). Albumin from human serum (A9511), fibrinogen from human plasma (F3879), and transferrin human (T3309) were purchased from Sigma-Aldrich. All other chemicals were purchased from Wako Pure Chemical Industries (Tokyo, Japan) and used as received. Water was purified and deionized using a Sartorius (arium 611 UV) water purification system (Sartorius AG, Goettingen, Germany). HeLa (RCB0007) and A549 (RCB0098) cell lines were obtained from the Riken Bio-Resource Center (Tsukuba, Japan). NIH3T3 fibroblast cell lines (ATCC^®^ CRL-1658) were obtained from ATCC (ATCC, Manassas, VA, USA).

### 2.2. Synthesis of SiO_2_-Embedded SiQD Powder

TES (16 mL) was placed in a two-neck round-bottom flask kept in an ice bath and stirred in an Ar atmosphere. Acidic water (32 mL, pH 3 using 12 M HCl) was added dropwise to TES with vigorous stirring under an Ar gas flow. The resultant Xerosol formed within 2 h. It was dried overnight in a vacuum. The dried white powder (i.e., amorphous hydrogen silsesquioxane) was placed in a quartz crucible, transferred to a high-temperature furnace, and heated at 1150 °C for 3 h in a flow of 5%/95%-H_2_/Ar. A uniform, dark-brown color solid powder was obtained. The powder was ground mechanically in a mortar to yield SiO_2_/SiQD powder samples.

### 2.3. Synthesis of a Free-Standing, Decane-Terminated SiQD

The SiO_2_/SiQD powder (300 mg) was placed in a small Teflon container and slurried in a mixture of ethanol (10 mL) and HF (48%, 10 mL). The mixture was stirred vigorously for 80 min, which gradually removed the SiOx matrix by acidic etching. The resulting hydrogen-terminated SiQDs were isolated by centrifugation at 15,000 rpm for 5 min and washed twice with ethanol, followed by isolation through centrifugation and decanting. The product was transferred to a Schlenk flask containing 1-decene. The resulting slurry was purged for at least 15 min with Ar at room temperature and subsequently heated at 170 °C for 2 h in an Ar atmosphere. The mixture was cooled to room temperature. Then the resulting decyl-coated silicon quantum dots (SiQDs-De) were further purified using high-performance liquid chromatography (HPLC). The purified SiQD-De was dried under vacuum conditions and redispersed in toluene.

### 2.4. Preparation of Pluronic-F127 Coated SiQDs-De

Pluronic F127 (200 mg) was dissolved in 10 mL of water containing 1 mL of 0.1 NaCl. The mixture was stirred for one h. A toluene solution (5 mL) containing 1 mg of SiQDs-De was gently poured into a vial of the Pluronic F127 solution. The vial was vigorously shaken for a couple of minutes to form an emulsion and was placed in a fume hood open to the air. After 48 h, the toluene layer of toluene evaporated. The water layer was sonicated for a few minutes and was subsequently transferred to a 14-KDa dialysis tube for around two h to remove the unreacted Pluronic F127 and HCl. The final sample was a milky solution. For biomedical applications, further dilution in water will be useful.

### 2.5. Characterization

UV-visible absorption spectra were recorded on a JASCO V-650 spectrophotometer (JASCO V-7200, Tokyo, Japan). The X-ray powder diffraction (XRD) pattern was acquired with a Rigaku Smart Lab X-ray diffractometer (Rigaku, Tokyo, Japan). Si-QDs sample’s crystalline lattice structure was observed with a high-resolution transmission electron microscope, HR-TEM, Tecnai G2 F30 (Field Electron and Ion Company, OR, USA) operated at 300 kV. For HR-TEM observations, an aqueous solution of the SiQD-De/F127 was dropped on an ultrathin-carbon (<10 nm thick) coated copper grid and dried under vacuum conditions before observations. PL and PL excitation (PLE) spectra of SiQD-De/F127 dispersed in Milli-Q water were measured using an InGaAs detector for NIR (Hamamatsu Photonics, Hamamatsu City, Japan) at room temperature on a NanoLog spectrofluorometer (Horiba Jovin Yvon, Tokyo, Japan) equipped with a 450 W xenon arc lamp. Protein samples are excited with 279 nm LED source/excitation wavelength. The solution sample’s time-resolved PL decay curves were acquired at room temperature on the same NanoLog Horiba Jovin Yvon spectrofluorometer in a multi-channel scaling (MCS) mode using a pulsed spectral LED of 370 nm as the excitation source. The curve-fitting quality for the experimental decay plots was assessed based on the χ^2^ value (~1.0) and visual inspections of the residuals. The absolute values of Photoluminescence Quantum Yield (PLQY) were measured by the standardized integral sphere method using a C9920-03G system equipped with a 150 W xenon lamp (Hamamatsu Photonics, Hamamatsu City, Japan). CD spectra of the plasma protein samples and the proteins complexed with SiQD-De/F127 were recorded with a spectropolarimeter (Jasco J-725, Tokyo, Japan) with 1 mm quartz cuvette at a scan speed of 100 nm/min at 25 °C. Each spectrum is an average of three scans. All the protein secondary structure predictions were calculated using a web server BESTSEL.

### 2.6. Asymmetric-Flow Field-Flow Fractionation (AF4) Instrumentation and Separation

The AF4 instrument consisted of an isocratic pump (1260 series (G1310B), Agilent Technologies, Santa Clara, CA, USA) attached to an HPLC manual injection valve (Wyatt Technology, high-performance injection system, Santa Barbara, CA, USA) with a 20 μL stainless steel sample loop, a field/flow control module, an AF4 separation channel (Eclipse, Wyatt Technology, Santa Barbara, CA, USA) fitted with a regenerated cellulose membrane (MW cutoff 5 kDa), a multiangle light scattering (MALS) detector (Dawn 8+, Wyatt Technology, Santa Barbara, CA, USA), and an ultraviolet-visible (UV-vis) absorbance diode array detector (1260 DAD (G1315D), Agilent Technologies, Santa Clara, CA, USA).

The AF4 analysis was performed using Phosphate Buffer Saline (PBS) (10 mM) as the eluent, filtered through a 0.22 µm Whatman filter, and sonicated before use. The analysis was carried out with a focusing/injection step, a 0.25 mL/min focusing-flow rate, and a 0.2 mL/min injection-flow rate. This was followed by an additional 2 min focusing/relaxation step. About 50 μL of the sample was injected into the system. When the sample was injected into the channel, axial flow and the focus flow opposed each other, concentrating the sample to a small area. The elution consisted of an initial equilibration with a cross-flow of 0.25 mL/min for about 10 min. The cross-flow gradually decreased to 0 over 10 min. The detector flow rate was maintained at 0.5 mL/min throughout the analysis. The AF4 analysis was carried out using the samples. The collection and analysis of data were performed using ASTRA software (Version 5.3.4.15, Wyatt Technology, Santa Barbara, CA, USA).

### 2.7. Cell Culture

HeLa, A549, and NIH3T3 cells were cultured in a 75 cm^2^ flask for the cytotoxicity assay and fluorescence microscopy observations. HeLa cells were maintained in a minimum essential medium added with fetal bovine serum (10%), penicillin (50 U/mL), and streptomycin (50 mg/mL) at 37 °C in humidified air containing 5% CO_2_. A549 cells and NIH3T3 cells were cultured in the same environments but using Dulbecco Modified Eagle Medium with low glucose.

### 2.8. Cytotoxicity Assay

Cell Counting Kit-8 (Dojindo Laboratories, Osaka, Japan) was used to measure the in vitro cytotoxic assay for the SiQD-De/F127 nanoparticles. In a 96-well plate, 5000 cells per well were seeded. After 24 h incubation at 37 °C, nanoparticles at various concentrations were added. Then 10-µL CCK-8 solution was added to each well and incubated for another 2 h. The absorbance was measured on a microplate reader (MTP-880Lab; Corona, Hitachinaka, Japan) at 450 nm. Finally, the cell cytotoxicity was indicated as a percentage compared with untreated control cells.

### 2.9. Cellular Uptake and Confocal Microscopy

To analyze the intracellular localization of the SiQD-De/F127 nanoparticles, we added nanoparticles at a final concentration of 100 µg/mL to NIH3T3 cells cultured in a 35-mm dish for 24 h. The cells were then washed thrice with PBS and fixed with 3.7% formaldehyde for 20 min. The differential interference contrast and fluorescence images were obtained with a confocal laser scanning fluorescence microscope (SP5, Leica Microsystems, Wetzlar, Germany) under a UV-LED laser. Besides, we observed the nanoparticles directly under a confocal microscope using the same laser source.

## 3. Results

### 3.1. Synthesis of a Water-Soluble Nanoparticle of SiQDs

**Scheme 1:**
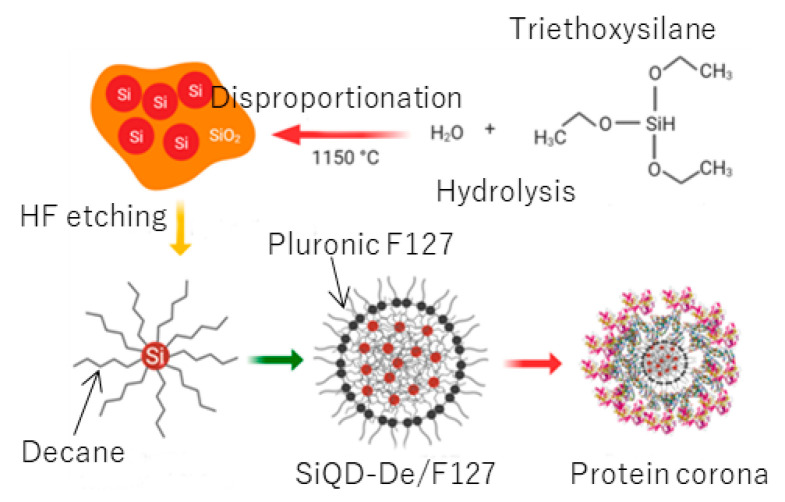
The schematic diagram for synthesizing the Pluronic-F127 functionalized decyl-coated silicon quantum dots (SiQD-De) nanoparticles and the nanoparticles’ protein corona formation assumption.

**Figure 1:**
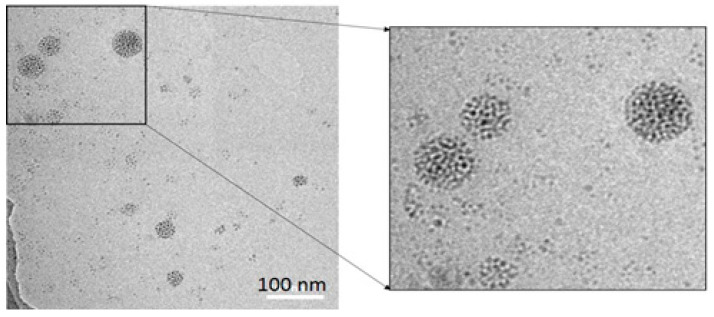
A typical TEM photograph of SiQD-De/F127 nanoparticles. The 40–70 nm diameter cores made up of assembled SiQDs-De (contrasted in the picture) are encapsulated with a Pluronic F127 molecule.

Following our previous protocol (Scheme 1) [37], we prepared a water-borne nanoparticle that consists of a Pluronic F127 shell, and a core made up of assembled SiQDs kept apart by decane monolayers (see Figure 1). Hydrogen-terminated QDs were prepared by thermal disproportionation of the hydrolysis product of TES and subsequent hydrofluoric acid etching. The QDs were composed of a diamond cubic lattice structure (Figure S1a, Supporting Information). The diameters of the QDs were controlled by optimizing the reaction temperature and hydrofluoric etching [38,39]. Next, the QDs were terminated with decane monolayers via thermal hydrosilylation of 1-decene to yield SiQD-De (Figure S1b, Supporting Information). Termination of SiQDs with alkyl monolayers drastically enhanced PLQYs [40,41,42]. The absolute values of PLQY measured by a standardized integrating sphere method were 40–45% in this work. The SiQD-De was transferred from toluene to water by encapsulation with a Pluronic F127 molecule (Figure S2, Supporting Information). The resultant SiQDs-De/F127 was purified by dialysis with a molecular weight cutoff (MWCO) 14 kDa tube.

We prepared four samples of SiQDs-De, which exhibited PL spectra peaking at 886 nm, 800 nm, 775 nm, and 725 nm (Figure S3, Supporting Information). The QD size tuned the peak position based on QC’s effect, consistent with the previous paper [37]. The absolute values of PLQY were 20–25%. The biological study employed the SiQD-De/F127 sample, which exhibited PL peaking at 725 nm and 25% QY. The SiQD-De assemblies had diameters of 30–100 nm and surface encapsulated with Pluronic F127 micelles (see Figure 1).

The micelle size in a buffer or culture medium is an essential parameter for biomedical use. Dynamic light scattering is a conventional technique for measuring the micelle size, but the size measured is biased towards a larger size. To address this, we used asymmetrical flow field-flow fractionation (AF4) coupled with static and dynamic light scattering (SLS and DLS) and fluorescence detectors to determine the size distribution of our nanoparticles of SiQD-De/F127. An advantage of AF4 analysis is that the size fractionation precedes the measurement, allowing the size distribution, including the multimodal one, to be precisely measured [36]. A typical AF4 fractogram of the SiQD-De/F127 sample shown in Figure S4 (Supporting Information) represents DLS’s elution profile. The fluorescence and DLS values coincided, confirming that the fluorescence signals originate from the micelles with Rh values of 70–100 nm, consistent with the TEM observation results.

### 3.2. Interaction Between Nanoparticles and Plasma Proteins—Steady-State Fluorescence

**Figure 2:**
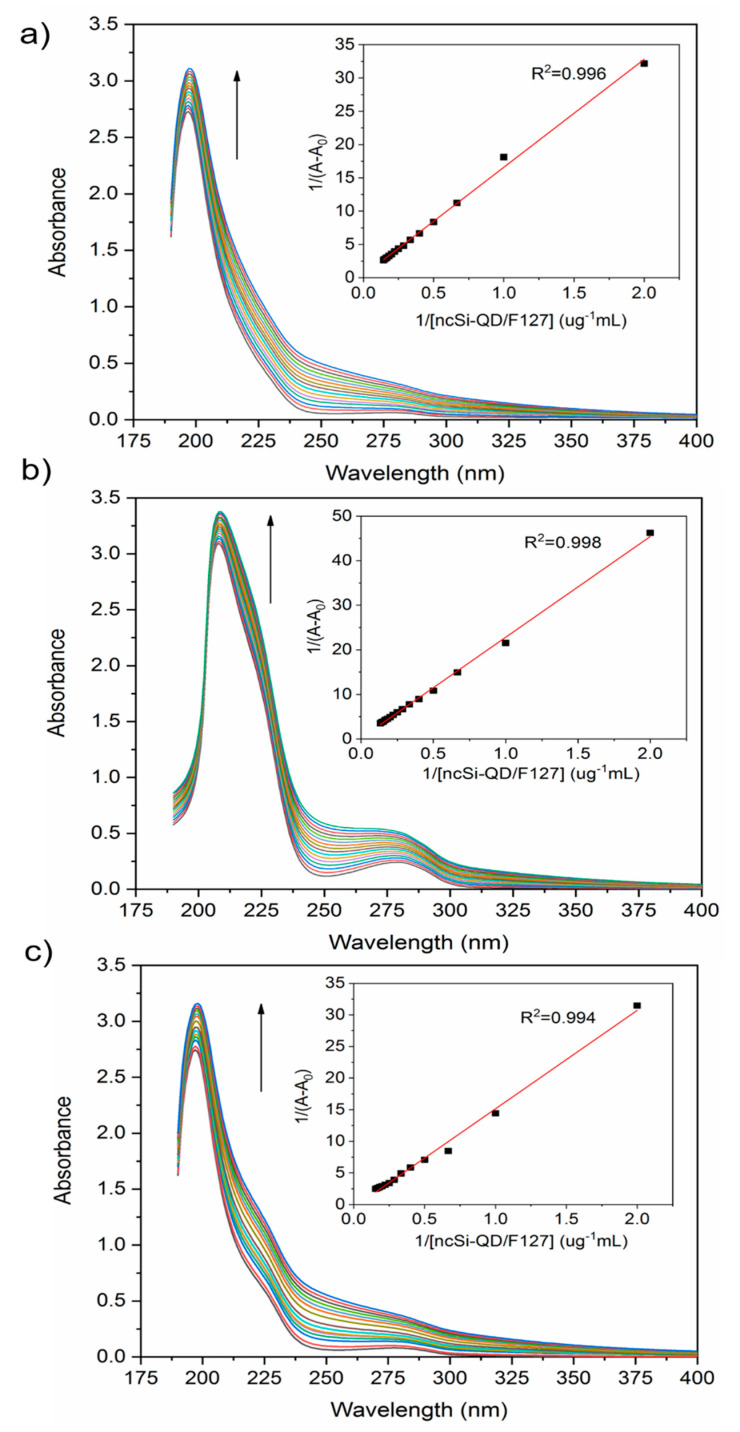
UV-visible absorption spectra of human serum albumin (**a**), fibrinogen (**b**), transferrin (**c**) with different concentrations (0–2.0 μg/mL) of SiQD-De/F127 nanoparticles and each proteins concentration are 1 µM. Optical absorbance increases continuously with the incremental addition of the nanoparticles. Insets show Benesi–Hildebrand plots for proteins as a function of nanoparticle concentration.

UV-vis spectrometry measured the molecular interaction between the SiQD-De/F127 nanoparticles and the plasma proteins. The changes in optical absorption behaviors of the proteins (1 µM) were recorded. The addition of nanoparticles (0–2 µg/mL) influenced the proteins’ spectral properties (albumin, fibrinogen, and transferrin) in the 190–400 nm range. Figure 2 shows the influence of the addition of nanoparticles. There were two absorption bands: the π-π * transition of the polypeptide backbone C=O of the proteins (λ_abs_ = ~190–230 nm) and the aromatic amino acid residues (λ_abs_ = ~280 nm). When the nanoparticles interacted with fibrinogen and transferrin, both absorption peaks, which appeared in the 190–230 nm range and around 280 nm, were red-shifted. However, the change in the peak positions due to albumin interaction was too small to be distinguished. The intensity of each absorption maximum was enhanced as the nanoparticle concentration increased (see Figure 2, inset). The changes in absorption were attributed to the emerging effect of a polar solvent, such as water, the perturbation of the microenvironment around the aromatic amino acid residues of proteins (i.e., tryptophan, tyrosine, and phenylalanine), and the ground state complex formation between the proteins and the nanoparticles as these lead to a shrinking energy gap of the π-π * transition [43]. Therefore, spectral redshifts were observed for the plasma proteins except for albumin.

**Figure 3:**
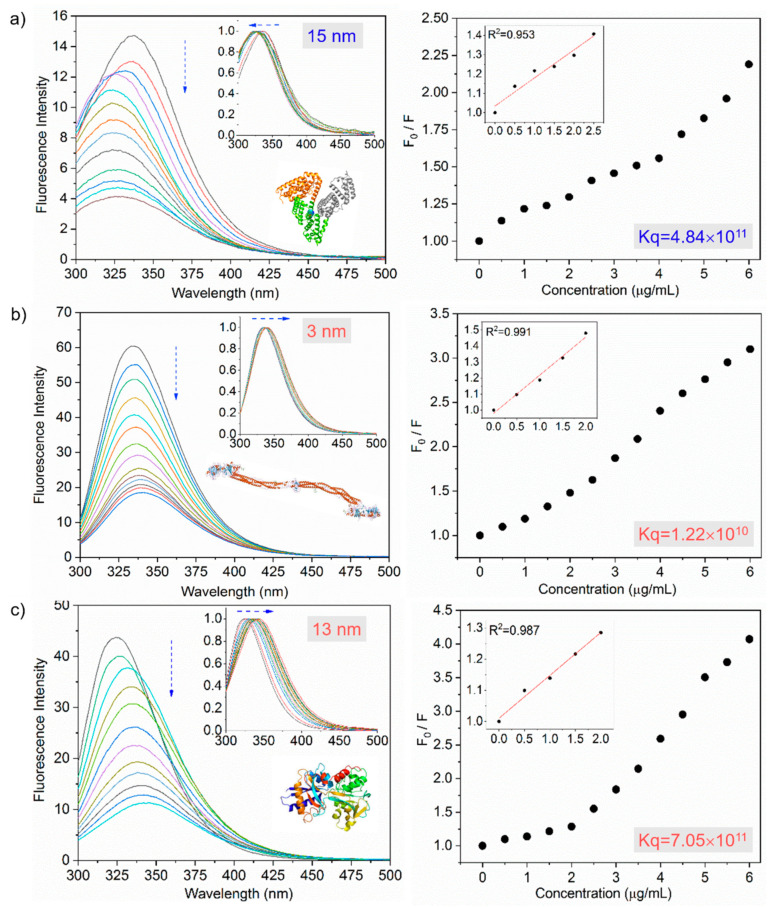
Fluorescence quenching (down arrow) of plasma proteins by SiQDs/F127 nanoparticles. Fluorescence emission spectra and its corresponding Stern–Volmer plots of human serum albumin (**a**), fibrinogen (**b**), and transferrin (**c**) in the absence and presence of SiQDs/F127 nanoparticles. The left side panel’s inset shows the spectral shift (horizontal arrows) of protein after interaction with the nanoparticles. The inset of the right-side panel shows the Stern–Volmer plots of proteins at lower concentrations after interaction with nanoparticles. Kq represents the quenching constant after the addition of nanoparticles. Protein crystal structures are taken from the protein data bank.

Similarly, the PL bands were also sensitive to microenvironmental changes of the aromatic amino acid residues. Conventionally, the PL spectrum of tryptophan is used in evaluations because its PL intensity is much stronger than other amino acid residues. The PL spectra demonstrate that the proteins’ intrinsic tryptophanyl fluorescence spectra are influenced by the nanoparticles’ incremental addition (see Figure 3). Tryptophan is known to exhibit PL spectral peaking at 309 nm in a fully buried configuration but 345–349 nm in a partially buried configuration [44,45]. For albumin, PL spectrum peaking at 338 nm, which was due to tryptophan buried in a hydrophobic interior of a protein, blue-shifted with the incremental addition of the nanoparticles, reaching 323 nm at 6.0 μg/mL nanoparticles. The blueshift of ~15 nm suggests that the tryptophan is buried in a more hydrophobic microenvironment, formed by protein aggregation. This is consistent with the PL behavior reported for bovine serum albumin (BSA) denatured by heat [46].

In contrast, the other three proteins exhibited continuous redshifts of the PL spectra with the nanoparticles’ incremental addition. Specifically, the PL spectral peaks at 335 nm, 324 nm, shifted to 338 nm, 337 nm for fibrinogen, transferrin, respectively. These redshift trends were attributed to the enhanced polarity of the aromatic acid residue tryptophan’s microenvironments, suggesting that they are exposed to the aqueous buffer upon interacting with the nanoparticles.

A redshift trend of the PL spectrum is a phenomenon that is typically observed in studies on heat-induced denaturation of proteins [47,48]. The large spectral redshift of ~13 nm implies a strong interaction between transferrin and the nanoparticles, leading to a great degree of exposure of the tryptophan moiety to the aqueous buffer phase and enhanced denaturation [49]. The observation of the fluorescence spectroscopic study is consistent with the results obtained by the optical absorption study.

All proteins displayed continuous decreasing trends in the PL intensity. PL quenching, a common phenomenon, originates from various molecular interactions between proteins and nanoparticles, such as excited-state reactions, molecular rearrangements, energy transfer, ground-state complex formation, and collisional quenching [50,51]. Strong fluorescence quenching implies the enhanced perturbations in the tertiary structure of proteins due to the binding of additives with the aromatic amino acid residues, which are more exposed toward the additives’ surface [52]. The fluorescence quenching behavior is conventionally described by the Stern–Volmer equation as [53]

F_0_/F = 1 + K_sv_ [SiQD-De/F127] = 1 + k_q_ τ_0_ [SiQD-De/F127]. (1)

F_0_ is the original fluorescence intensity, and F is the tryptophanyl fluorescence intensity with the incremental addition of the SiQD-De/F127 nanoparticles as a quencher. K_sv_, k_q_, (SiQD-De/F127), and τ_0_ represent the Stern–Volmer constant, the biomolecular quenching rate constant, the quencher concentration, and the average lifetime of the original PL, respectively. Table 1 summarizes the calculated parameters. For all four proteins, a linear relationship between the fluorescence intensity and nanoparticle concentration was observed in a low concentration range (<2.0 µg/mL), indicating that the quenching in the lower concentration is dominated by static quenching (or ground state complex formation) (see Figure 3, inset) [50,51]. On the other hand, the PL quenching in the high concentration might be dominated by dynamic (or collisional) quenching [54]. According to Hadjidemetriou et al. [28], an inner layer of tightly bound proteins is called ‘hard corona,’ and an outer rapidly exchanging layer of weakly bound proteins is called ‘soft corona.’ In their work, both albumin and transferrin exhibit higher (10^11^th order) quenching constants, leading to hard protein corona formation with a stronger association. On the other hand, fibrinogen exhibit lower (10^10^th order) quenching constants, forming soft protein corona. Their micelle surfaces directly interacted with hard corona proteins; at the same time, soft protein corona associates with hard protein corona via weak protein-protein interactions.

**Figure 4:**
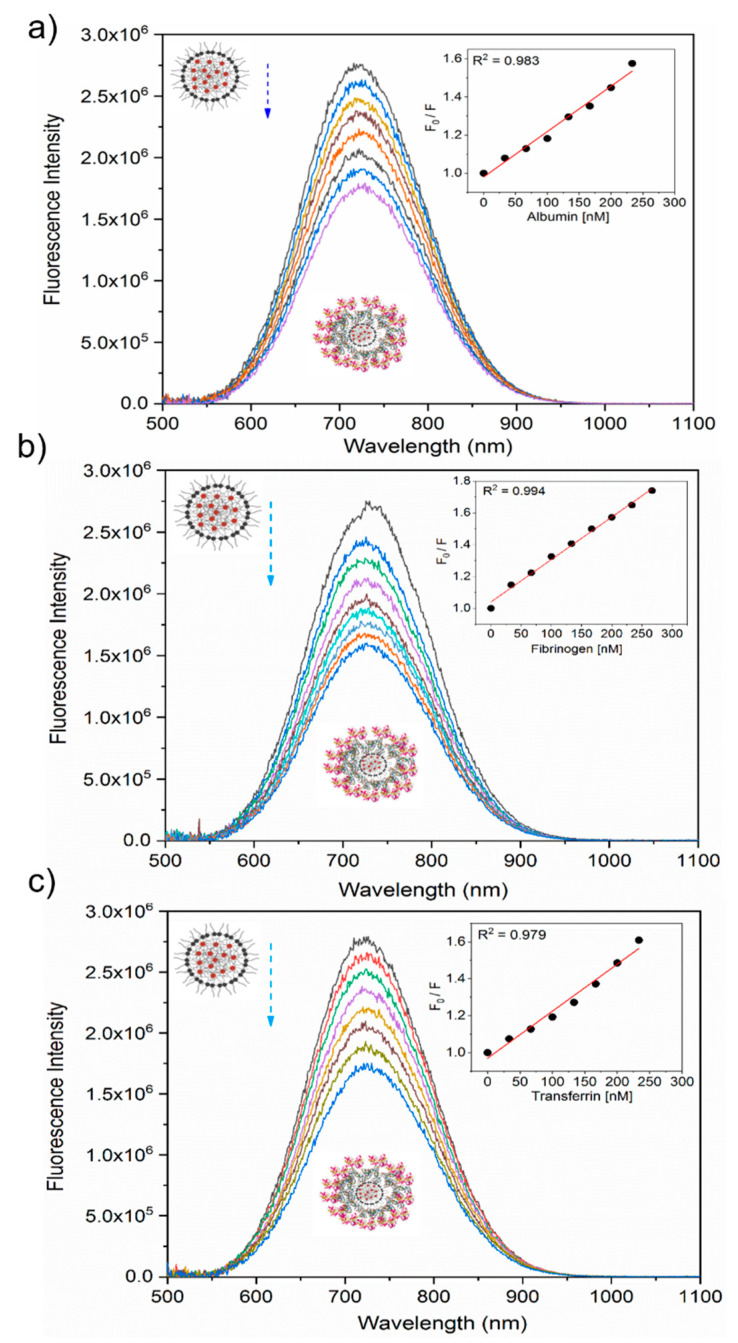
Photoluminescence (PL) spectra of SiQDs/F127 nanoparticles with the incremental addition of plasma proteins. Fluorescence emission spectra and its corresponding Stern–Volmer plots of SiQDs/F127 nanoparticles are shown in the absence and presence of human serum albumin (**a**), fibrinogen (**b**), and transferrin (**c**).

Next, we observed the nanoparticles’ fluorescence quenching nature with the incremental addition of plasma proteins (0–300 nM). Figure 4 shows the results, where the inset is the Stern–Volmer plots. The R^2^ values expressed the goodness of fit. The high value (>0.97) indicates linear quenching trends upon the addition of proteins for each interaction, implying that protein adsorption degrades the PL intensity of the nanoparticles. Table S1 (Supporting Information) tabulates the calculated parameters. The K_q_ values are below the upper limit of diffusion (2.0 × 10^10^ M^−^^1^s^−^^1^), indicating that the nanoparticles’ fluorescence quenching is dominated by the dynamic collisional process [53].

### 3.3. Interaction between Nanoparticles and Plasma Proteins—Excited State Fluorescence

**Figure 5:**
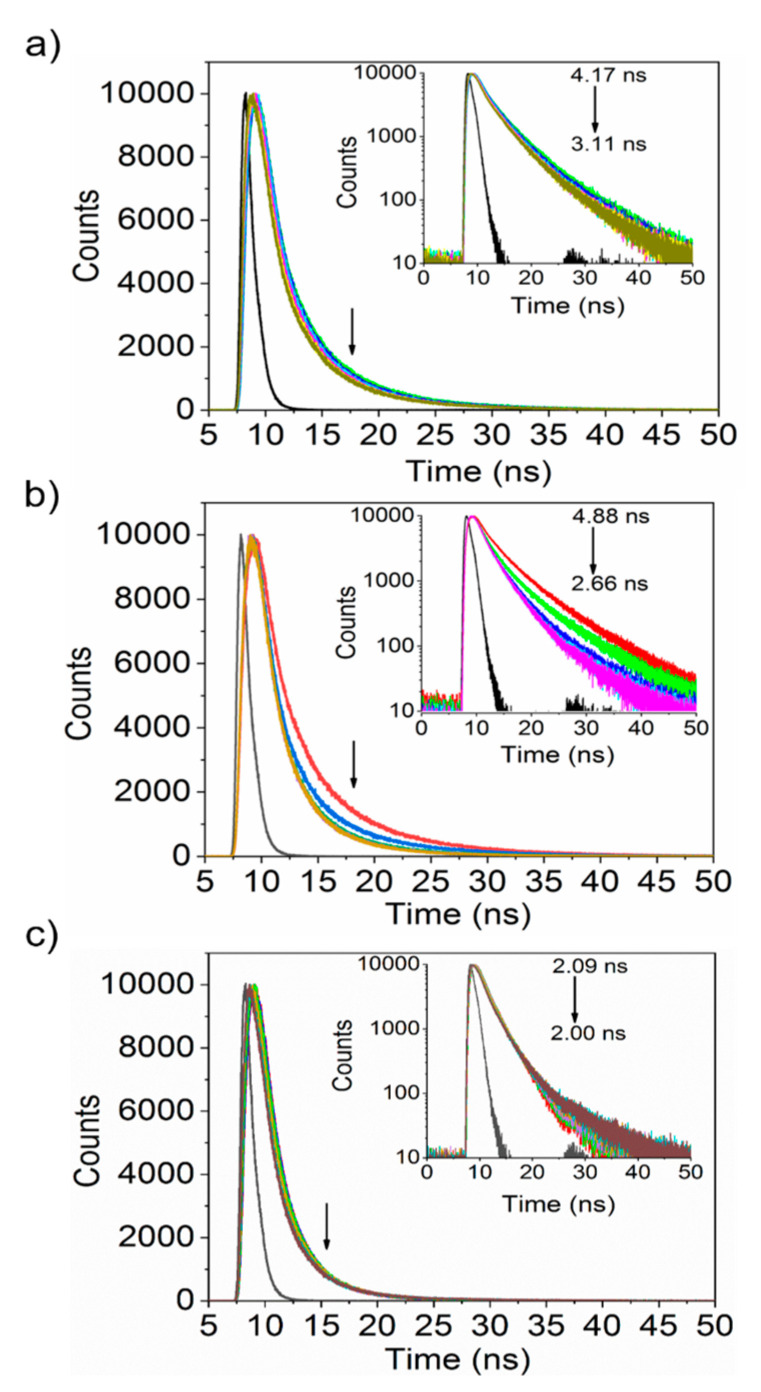
PL decay profiles of human serum albumin (**a**), fibrinogen (**b**), and transferrin (**c**) with the incremental addition of SiQDs/F127 nanoparticles. Arrows indicate the decrease of PL decay time with the gradual addition of nanoparticles.

To clarify the PL dynamics of the protein-nanoparticle complex, the PL decay curves were measured for the four plasma proteins with incremental nanoparticle concentration changes. Similarly, the PL decay curves for the nanoparticles were measured with gradual changes in the protein concentration. Figure 5 plots the decay curves of the proteins, which are approximately fitted with a triexponential function expressed by Equation (2). Table S2 (Supporting Information) summarizes the estimated decay time.

(2) $$I\left(t\right)\approx B_{1}exp\left[-\frac{t}{\tau_{1}}\right]+\;B_{2}exp\left[-\frac{t}{\tau_{2}}\right]+\;B_{3\;}exp\left[-\frac{t}{\tau_{3}}\right],$$

where τ_1_, τ_2_, and τ_3_ are the first, second, and third components of the PL lifetime, and B_1_, B_2_, and B_3_ are the amplitudes of each component, respectively. Increasing the nanoparticle concentration decreased the radiative lifetime (arrows in Figure 5, inset). For albumin, the decay curve was fitted with a triexponential function with time constants of 1.13, 0.12, and 5.31 ns, giving a calculated average decay time of 4.17 ns. In the presence of 13.33 µg/mL nanoparticles, the average lifetime decreased to 3.23 ns. Similarly, the values of average decay time for fibrinogen, transferrin decreased from 4.87 to 2.66 ns, from 2.08 to 2.10 ns, respectively. The insignificant differences in tryptophanyl PL decay time indicate that nanoparticles’ addition does not affect the PL decay dynamics.

**Figure 6:**
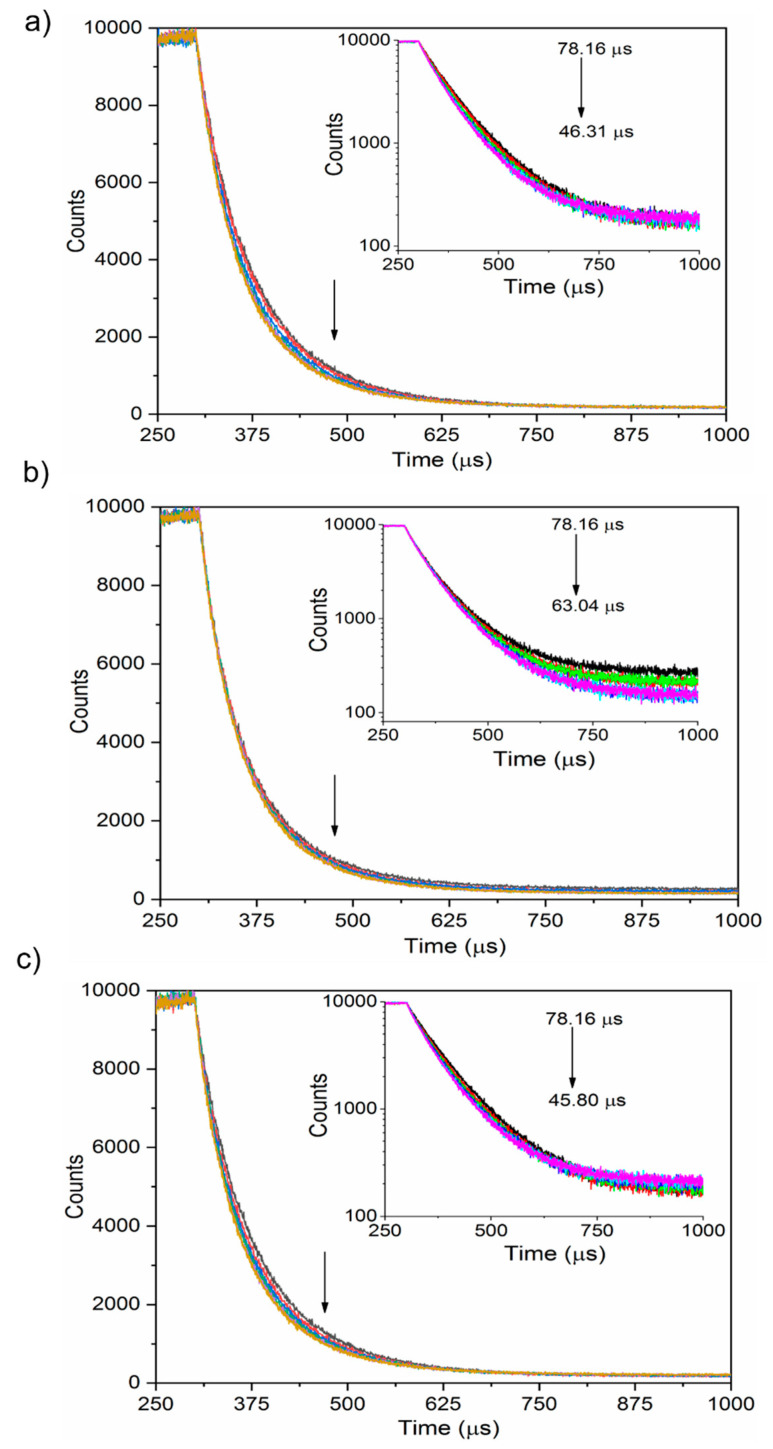
PL decay profiles of SiQDs/F127 nanoparticles with the incremental addition of blood plasma proteins (down arrow), human serum albumin (**a**), fibrinogen (**b**), and transferrin (**c**). Insets show the fluorescence lifetime of nanoparticles after interaction with proteins.

The PL decay profiles measured for the nanoparticles with the incremental addition of proteins were fitted with a biexponential function (Figure 6). Table S3 (Supporting Information) provides the fitting parameters. All the proteins showed decreasing trends for the decay time as the concentration increased (arrows, Figure 6, inset). For the albumin, an average decay time of 78.1 µs was estimated using a bi-exponential fit with lifetime components of 26.1 and 80.3 µs. After the serial addition of albumin, the average lifetime of the nanoparticles decreased to 46.3 µs. Similarly, the other two proteins also exhibited decreasing trends of the average decay time with an incremental protein concentration ranging from 78.1 to 63.0 µs for fibrinogen, as well as from 78.1 to 45.8 µs for transferrin. Unlike the PL dynamics of the tryptophanyl fluorescence, the proteins’ incremental addition influenced the nanoparticles’ PL decay time.

Considering the dramatic reduction of PLQY after Pluronic F127 encapsulation, the nanoparticles’ decreased PL decay time is discussed as follows. PLQY (η) is expressed as η = k_r_/(k_r_ + k_nr_), where k_r_ = 1/τ_r_ and k_nr_ = 1/τ_nr_ (τ is the characteristic PL lifetime) using two parameters of k_r_ for radiative and k_nr_ for nonradiative processes. Adsorption of proteins to the nanoparticle surface disturbs the molecular configuration of the Pluronic F127 micelle. This may lessen the micelle’s blocking performance against water molecules and oxygens, leading to slight oxidation of QDs. The oxidized QD surface works as a nonradiative channel to increase the value of τ_nr_ [55], leading to a shorter PL decay time.

### 3.4. Protein Secondary Structure Alteration/Recovery upon Interaction with Nanoparticles

A detailed understanding of the secondary-or tertiary-structural changes of proteins upon binding with concentrated nanoparticles is necessary to appreciate the mutual interaction, realizing practical improvements in biocompatibility. Protein-nanoparticle interactions are common phenomena, which result in conformational changes or denaturation of the proteins adsorbed to the nanoparticles [56]. Investigations of such conformational changes have revealed that the protein can realize regular biological activity even after the interaction. CD spectroscopy is a convenient tool for this purpose. The secondary structures can be calculated using BeStSel developed for the secondary structure determination and folding recognition from protein CD spectra [57,58].

**Figure 7:**
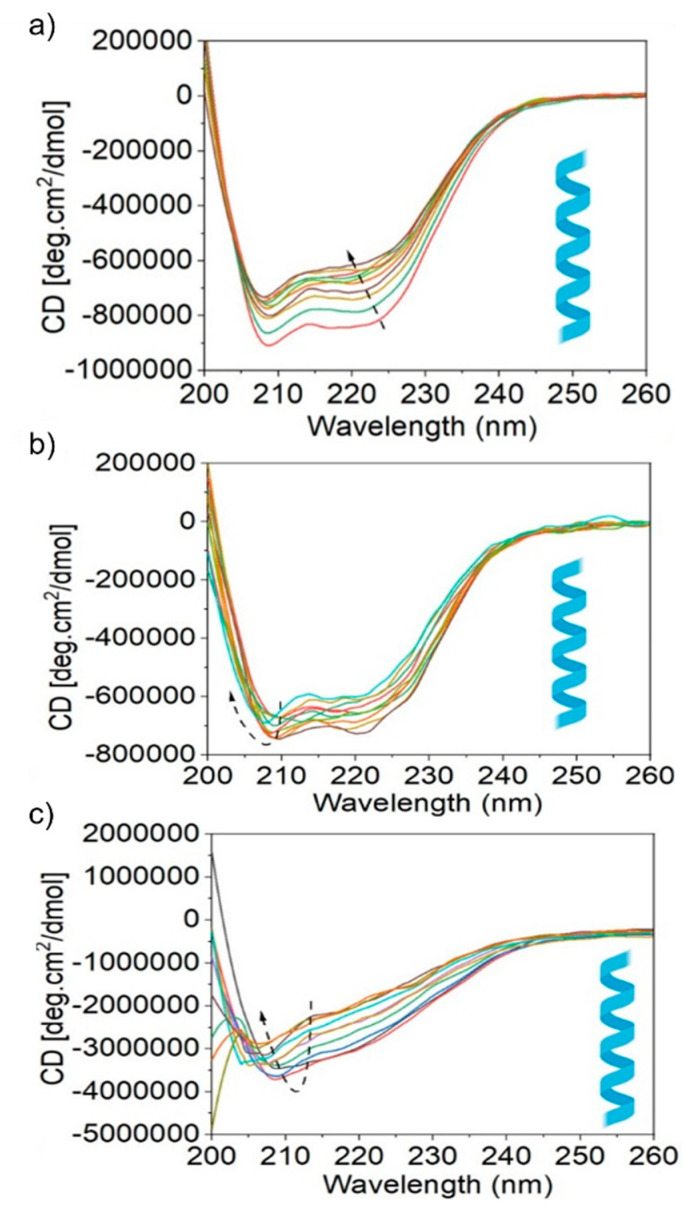
Circular dichroism (CD) spectra are demonstrating the secondary structure and conformational stability of the human blood plasma proteins albumin (**a**), fibrinogen (**b**), and transferrin (**c**) upon association with SiQDs/F127 nanoparticles (in the 0–2.25 µg/mL concentration range at 0.25 intervals). Arrows indicate the nature of spectral shift or change after the addition of nanoparticles with proteins.

Figure 7 shows the UV CD spectra of the four proteins measured between 200 and 260 nm with the nanoparticles’ incremental addition. Figure 7a–c show two bands around 208 and 222 nm due to albumin, fibrinogen, and transferrin with the α-helical secondary structure, respectively. The shapes and positions of the peaks were altered upon the addition of 1 µg/mL nanoparticles, indicating the onset of protein denaturation. Native albumin (0.5 µM) contained 65.80% regular α-helical structure, but the value gradually decreased to 50.73% by the incremental addition of nanoparticles (in the 0–2.25 µg/mL concentration range at 0.25 intervals) (see Table S4, Supporting Information). The α-helix changes of proteins are minimal (15%) upon interaction with nanoparticles; it does not affect the protein’s tertiary structure.

For fibrinogen (see Table S5, Supporting Information), the regular α-helical structure (19.94%) decreased to 6.74% upon adding 1.25 µg/mL nanoparticles. Surprisingly, the decreasing trend changed, and at a 2.25% nanoparticle addition, the percentage of the α-helical structure approached the original position. Transferrin showed a similar trend for the α-helical characteristics (see Table S6, Supporting Information). In this case, a regular α-helical structure (26.14%) decreased continuously to 10.81% with the nanoparticles’ incremental addition. However, it turns an increasing trend at 1.25 µg/mL nanoparticle concentration and reached a 25.06% regular α-helical structure close to the original percentage.

Plasma proteins and their interaction with quantum dots are reported in various physiological conditions [59,60,61]. Recently, Hui reported CdTe QDs interaction with human serum albumin (HSA), metallothionein-II TM, FIB, and PLG [61]. However, the secondary structural changes in the proteins are not studied clearly. Most of the experiments were conducted with few QDs concentrations with proteins. We showed extended concentrations of the QDs to observe the secondary structure recovery phenomenon. To the best of our knowledge, such a recovery phenomenon has not yet to be reported for the interaction between the plasma proteins and additives, including QDs. However, a few papers have reported the heat-induced conformational change and recovery of proteins [62]. For practical bioapplications, including drug delivery, protein-modified SiQD biomarkers must be suitable in terms of biocompatibility.

### 3.5. Cellular Uptake and Cytotoxicity of Nanoparticles

In addition to the above interaction studies, we demonstrated the nanoparticles’ cellular uptake and cell cytotoxicity to show biocompatibility. The SiQDs-De/F127 nanoparticles’ cellular toxicity was investigated using three representative cell lines: HeLa, A549, and NIH3T3 (fibroblast) cells (Figure S5, Supporting Information). The average values in the viability of Hela, A549, and NIH3T3 cells remain ~100% throughout 24 h incubation, which is a conventional condition. Even 100 µg/mL of SiQD-De/F127 nanoparticles did not display cytotoxicity for all the cells. Under more harsh cell conditions (i.e., 48 h incubation), the nanoparticles were non-toxic to both HeLa and NH3T3 cells. The viability of A549 cell decreases down to ~80%, but its toxicity is significantly less than the heavy-metal QDs [18].

**Figure 8:**
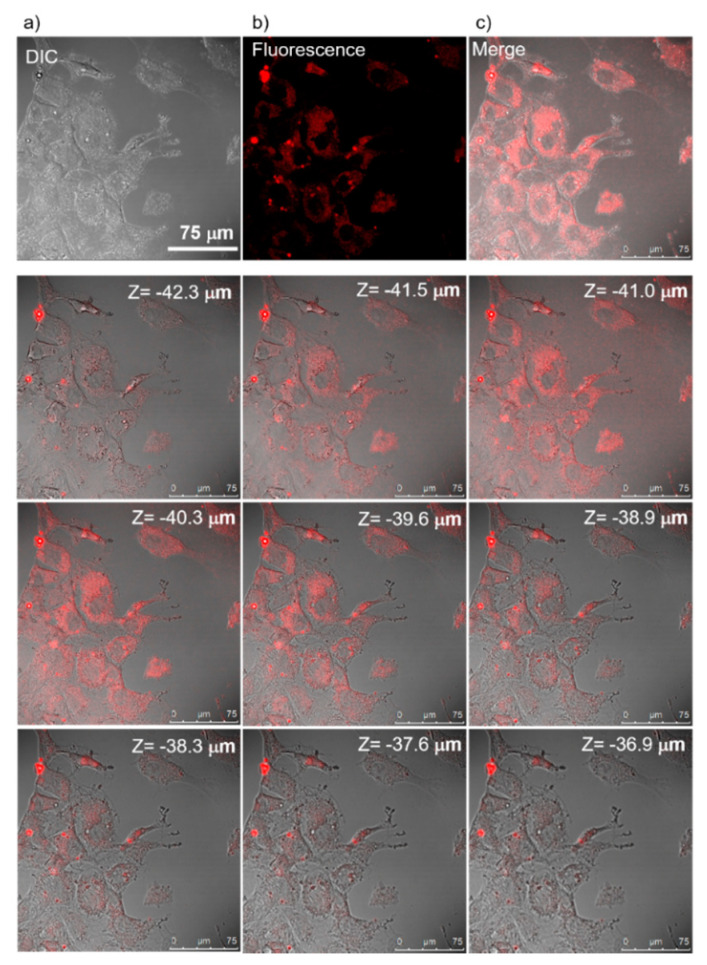
NIH3T3 (Fibroblast) cellular uptake of SiQDs/F127 nanoparticles under confocal microscopy. (**a**) Differential interference contrast (DIC), (**b**) fluorescence, and (**c**) overlap images. Slice scan of NIH3T3 cells under confocal microscopy.

Further study is necessary to clarify the mechanism of the appearance of the toxicity to A549 cells. To compare with A549 cells, the NIH3T3 fibroblast cells are much safer even at higher nanomaterial uptake concentrations. Since the accuracy of nontoxicity (~100% cell viability at 100 mg/mL nanoparticle concentration) should be confirmed, a fluorescence confocal microscope was used to observe the culture’s uptake cells. NIH3T3 fibroblast cells were imaged by fluorescence contrast (see Figure 8). A consecutive sectional scan of mouse fibroblast cells along the Z-direction confirmed the nanoparticles’ cellular uptake. The nanoparticles were distributed throughout the cytosol and did not enter the cell nucleus.

## 4. Conclusions

We synthesized the NIR-emitting, biocompatible SiQDs/F127 nanoparticles (25% of PLQY at pH 7 in water) for a detailed investigation of the nanoparticles’ molecular compatibility. We used a few major human blood plasma proteins (albumin, fibrinogen, and transferrin) for this purpose. First, we investigated optical absorption and emission properties of proteins with the incremental addition of nanoparticles and vice versa. Interestingly, plasma proteins’ binding parameters depended on the interaction between the nanoparticles and the protein binding pockets or surface. Specifically, albumin and transferrin had higher (10^11^th order) quenching constants. They formed a hard protein corona with a stronger association.

On the other hand, fibrinogen had lower (10^10^th order) quenching constants, forming a soft protein corona. The fluorescence spectral shift of the proteins upon binding with the nanoparticles confirmed the proposed protein corona concept. The CD spectroscopic study suggests that SiQD-De/F127 nanoparticles have a potential for medical diagnosis without major conformational change of proteins. In addition, to support the above statements, we observed enabled cellular uptake (NIH3T3) and minimal cytotoxicity using different cell lines (HeLa, A549, and NIH3T3). This study offers a basis to design QDs without altering biomacromolecules’ original conformation with enabled cellular uptake with minimal cytotoxicity. This work might contribute to the design of new biomarkers for in-vivo towards biological applications.

## Figures and Tables

**Table 1:** The Stern–Volmer constant (KSV), biomolecular quenching rate constant (kq), lifetime (τ_0_), and the number of the binding site (n) for four classes of proteins interacted with the SiQD-De/F127 nanoparticles. R^2^ shows a good fit with Equation (1).

Protein	K_sv_ (ug^−1^ mL)	K_q_ (ug^−1^ mLs^−1^)	τ_0_ (ns)	n	R^2^
Albumin	0.20 ± 0.06	4.84 × 10^11^	4.17	1.03	0.95
Fibrinogen	0.59 ± 0.09	1.22 × 10^10^	4.88	0.99	0.99
Transferrin	0.15 ± 0.08	7.05 × 10^11^	2.09	0.95	0.95

## Supplementary Materials

The following are available online at https://www.mdpi.com/2079-4991/10/11/2250/s1, Figure S1: (a) A typical XRD spectrum of hydrogen-terminated SiQDs by HF etching for 85 min. (b) ATR-FTIR spectra of hydrogen-terminated SiQDs before and after hydrosilylation of 1-decence. Figure S2: Three different stages for preparing SiQD-De/F127 nanoparticles are demonstrated. Three pairs of photographs are taken under room illumination and UV light for each stage. Twenty-four microliters toluene of SiQD-De was poured into the glass bottle with 8 mL Milli-Q water. The amounts of toluene and water are indicated by arrows in a left-pair image. Figure S3: UV-visible absorption spectra, PL excitation (PLE) spectra, and PL spectra of four SiQD-De/F127 samples with different QD sizes. The diameters estimated from Scherrer broadening analysis are 4.8 nm, 3.4 nm, 2.9 nm, and 2.3 nm, respectively. Both PLE spectra centered at 328, 331, 352, 369 nm, and their corresponding PL spectra centered at 886, 800, 775, 720 nm spectrum are demonstrated for four SiQDs/F127 samples. Figure S4: AF4 fractogram with detection by DLS (red dots) of the nanoparticles in water. Figure S5: Typical results of cell viability after 24 and 48 h incubation with SiQD-De/F127 using (a) HeLa cells (b) A549 cells, and (c) NIH3T3 cells. Table S1: Binding parameters of SiQD-De/F127 nanoparticles during interaction with proteins. The Stern–Volmer constant (KSV), biomolecular quenching rate constant (kq), NPs lifetime (τ_0_), and the number of the binding site (n) for four classes of proteins interacted with the SiQD-De/F127 nanoparticles. R^2^ shows a good fit with the Equation (1). Table S2: Changes in tryptophanyl fluorescence lifetime of the proteins by incremental addition of the SiQD-De/F127 nanoparticles. Table S3: Changes in PL lifetime of SiQD-De/F127 nanoparticles by the gradual addition of the proteins. Table S4: Secondary structural analysis of albumin during interaction with SiQD-De/F127 nanoparticles. Table S5: Secondary structural analysis of fibrinogen during interaction with SiQD-De/F127 nanoparticles. Table S6: Secondary structural analysis of transferrin during interaction with SiQD-De/F127 nanoparticles.
