# Supplementary material for: Nano-Bio Interaction between Blood Plasma Proteins and Water-Soluble Silicon Quantum Dots with Enabled Cellular Uptake and Minimal Cytotoxicity
Source: Nanomaterials (Basel). 2020 Nov 13;10(11):2250. doi: 10.3390/nano10112250 (PMC7696914; doi:10.3390/nano10112250)
Supplement: Supplementary file 1 [file nanomaterials-10-02250-s001.pdf]

Supporting Information

Nano-Bio Interaction Between Blood Plasma Proteins and Water-Soluble Silicon Quantum Dots with Enabled Cellular Uptake and Minimal Cytotoxicity

Shanmugavel Chinnathambi [1,2,*], Nobutaka Hanagata [3,4], Tomohiko Yamazaki [4,5] and Naoto Shirahata [4,6,7*]

[1] International Center for Young Scientists, National Institute for Materials Science (NIMS), 1-2-1 Sengen, Tsukuba, Ibaraki 305-0047, Japan
[2] Institute for Integrated Cell-Material Sciences (ICeMS), Institute for Advanced Study, Kyoto University, Kyoto 606 8501, Japan
[3] Nanotechnology Innovation Station, NIMS, 1-2-1 Sengen, Tsukuba, Ibaraki 305-0047, Japan; hanagata.nobutaka@nims.go.jp
[4] Graduate School of Chemical Sciences and Engineering, Hokkaido University, Sapporo 060-0814, Japan, yamazaki.tomohiko@nims.go.jp
[5] Research Center for Functional Materials (RCFM), NIMS, 1-2-1, Sengen, Tsukuba 305-0047, Japan
[6] International Center for Materials Nanoarchitectonics (WPI-MANA), NIMS, Namiki, Tsukuba 305-0044, Japan
[7] Department of Physics, Chuo University, 1-13-27 Kasuga, Bunkyo, Tokyo 112-8551, Japan
* Correspondence: chinnathambi.shanmugavel.8s@kyoto-u.ac.jp (S.C.); shirahata.naoto@nims.go.jp (N.S.)

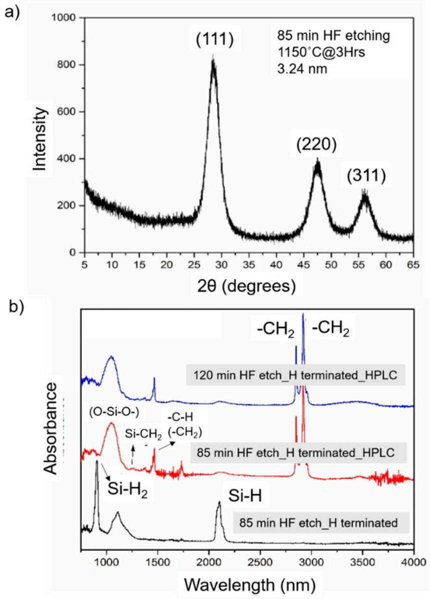

Figure S1. (**a**) A typical XRD spectrum of hydrogen-terminated SiQDs by HF etching for 85 min. (**b**) ATR-FTIR spectra of hydrogen-terminated SiQDs before and after hydrosilylation of 1-decence.

Note: The XRD pattern shows three peaks at 2θ = 28°, 47°, and 56° corresponding to the (111), (220), and (311) planes of the diamond cubic lattice structure of Si as shown in Panel (a). ATR-FTIR spectra of SiQDs before and after hydrosilylation are shown in Panel (b). The disappearance of Si-H vibrations at 2105 cm^{-1} and 910 cm^{-1} was observed during the hydrosilylation reaction. Instead, we observed the appearance of C–H stretching and bending/scissoring bands at around 2850–2960 cm^{-1} and 1350–1500 cm^{-1}, resulting in the termination with decane monolayers.

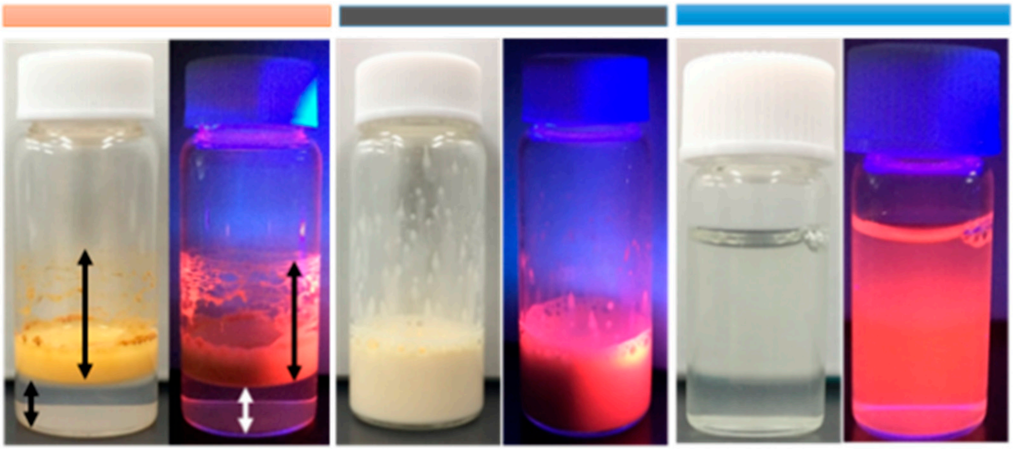

Figure S2. Three different stages for preparing SiQD-De/F127 nanoparticles are demonstrated. Three pairs of photographs are taken under room illumination and UV light for each stage. Twenty-four microliters toluene of SiQD-De was poured into the glass bottle with 8 mL Milli-Q water. The amounts of toluene and water are indicated by arrows in a left-pair image.

The left-pair image was taken after the evaporation of toluene in fume-hood. As clearly seen in the left image, a layer of SiQD-De (a yellow-color band) is left on the top of the water. Some of the SiQD-De are left inside the wall of the bottle. The red-fluorescence in the right image of the pair confirms that the yellow-color band consists of SiQD-De.

The middle-pair was obtained by shaking the glass bottle shown in the left-pair. The left image indicates that the milky-colored water solution of Si-De/F127. The right image indicates the well-dispersion of SiQD-De/F127 in the milky colored solution.

The right pair was obtained by dilution of the middle pair with Milli-Q water. The left image indicates a colorless and transparent water solution. The right image shows that SiQD-De/F127 nanoparticles are highly dispersed in water due to successful encapsulation of SiQD-De with Pluronic F127 molecule.

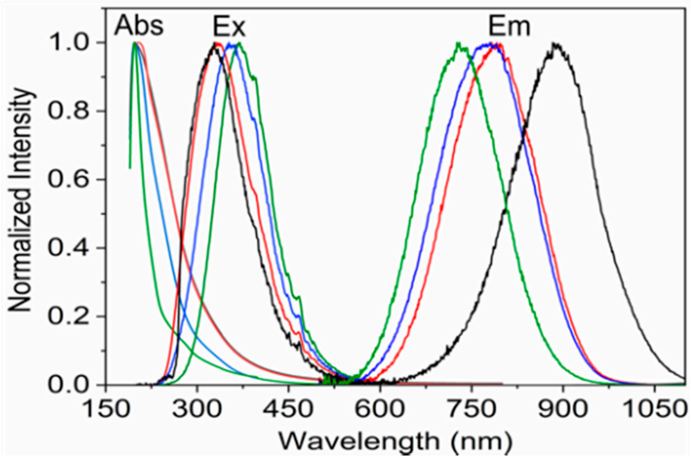

Figure S3. UV-visible absorption spectra, PL excitation (PLE) spectra, and PL spectra of four SiQD-De/F127 samples with different QD sizes. The diameters estimated from Scherrer broadening analysis are 4.8 nm, 3.4 nm, 2.9 nm, and 2.3 nm. Both PLE spectra centered at 328, 331, 352, 369 nm, and their corresponding PL spectra centered at 886, 800, 775, 720 nm spectrum are demonstrated for four SiQDs/F127 samples.

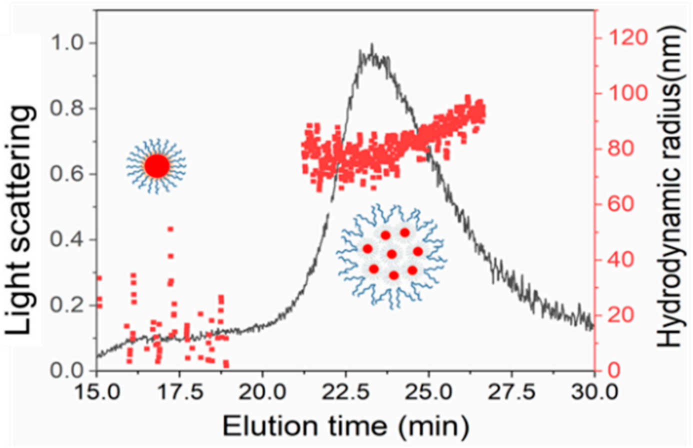

Figure S4. AF4 fractogram with detection by DLS (red dots) of the nanoparticles in water.

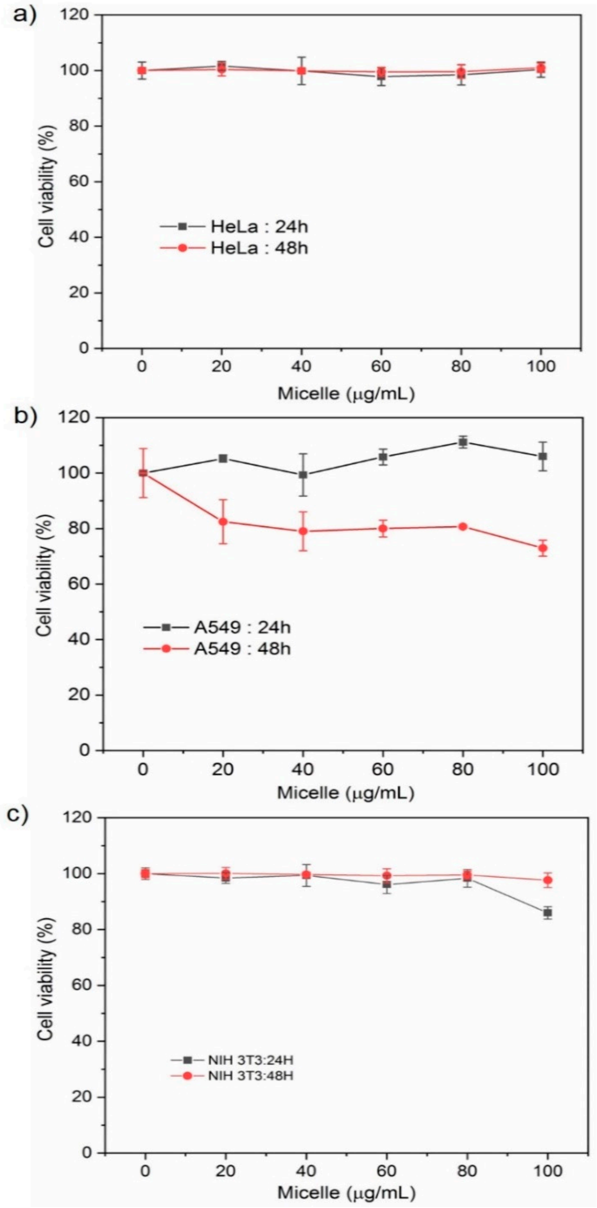

Figure S5. Typical results of cell viability after 24 and 48 h incubation with SiQD-De/F127 using (**a**) HeLa cells, (**b**) A549 cells, and (**c**) NIH3T3 (fibroblast) cells.

Table S1. Binding parameters of SiQD-De/F127 nanoparticles during interaction with proteins. The Stern–Volmer constant (K_{SV}), biomolecular quenching rate constant (k_q), NPs lifetime (τ_0), and the and the number of the binding site (n) for four classes of proteins interacted with the SiQD-De/F127 nanoparticles. R2 shows a good fit with the Equation (1).

**Nanoparticle/Protein.**	**K_{sv} ($L^{-1}M^{-1}$)**	**K_q ($L M^{-1}$)**	**τ_0 (μs)**	**n**	**R^2**
Nanoparticle/Albumin	0.221×10^7	2.83×10^{10}	78.16	0.901	0.985
Nanoparticle/Fibrinogen	0.318×10^7	7.79×10^{10}	78.16	0.947	0.994
Nanoparticle/Transferrin	0.219×10^7	3.09×10^{10}	78.16	0.904	0.979

Table S2. Changes in tryptophanyl fluorescence lifetime of the proteins by incremental addition of the SiQD-De/F127 nanoparticles.

	**Nanoparticle Conc. (ug/mL)**	**$\alpha_1 \times 10^3$**	**α_2**	**α_3**	**τ_1 (ns)**	**τ_2 (ns)**	**τ_3 (ns)**	**τ_0 (ns)**	**χ^2**
Albumin	0 (Only protein)	5.47	5.39×10^3	3.52×10^3	1.13	0.12	5.31	4.17	1.12
	03.33	5.60	8.76×10^3	3.56×10^3	1.08	0.08	4.84	3.74	1.11
	06.66	5.54	7.12×10^2	3.49×10^3	1.08	0.02	4.66	3.49	1.07
	10.00	5.35	5.71×10^2	3.52×10^3	1.07	0.03	4.49	3.34	1.08
	13.33	4.99	7.90×10^2	3.35×10^3	1.09	0.03	4.49	3.23	1.06
Fibrinogen	0 (Only protein)	3.19	4.28×10^3	1.66×10^3	3.32	1.02	7.54	4.87	1.06
	03.33	3.08	6.18×10^3	1.02×10^3	3.03	0.96	7.55	4.00	1.13
	06.66	3.64	6.24×10^3	8.18×10^4	2.75	0.86	6.33	3.15	1.11
	10.00	5.97	7.38×10^4	4.02×10^3	2.70	0.82	5.78	2.99	1.10
	13.33	2.71	2.42×10^3	5.23×10^3	1.72	0.74	3.14	2.66	1.09
Transferrin	0 (Only protein)	5.74	3.23E-02	4.06×10^3	9.92	0.03	2.82	2.08	1.03
	03.33	5.53	0.147133	4.02×10^3	9.76	0.01	2.89	2.08	1.05
	06.66	5.58	2.42E-02	3.53×10^3	1.09	0.06	3.05	2.16	1.07
	10.00	5.27	0.115876	3.32×10^3	1.08	0.02	3.14	2.09	1.12
	13.33	4.96	9.32E-02	2.90×10^3	1.13	0.03	3.33	2.10	1.13

Table S3. Changes in PL lifetime of SiQD-De/F127 nanoparticles by incremental addition of the proteins.

	**Protein Conc. (nM)**	**$\alpha_1 \times 10^3$**	**$\alpha_2 \times 10^3$**	**τ_1 (μs)**	**τ_2 (μs)**	**τ_0 (μs)**	**χ^2**
Albumin	0 (Only micelle)	1.07	8.44	26.1	80.3	78.1	0.97
	33.33	1.20	8.39	24.1	78.6	76.3	0.96
	66.66	1.31	8.05	24.4	74.3	71.7	0.99
	99.99	1.61	7.88	22.9	72.6	69.5	0.98
	133.32	1.15	8.26	19.9	66.3	64.4	0.99
	166.65	2.25	7.84	18.3	49.3	46.3	1.14
Fibrinogen	0 (Only micelle)	1.07	8.44	26.1	80.3	78.1	0.97
	33.33	2.80	6.16	32.1	78.0	70.7	0.95
	66.66	2.98	6.30	30.1	76.3	69.0	1.01
	99.99	2.17	7.37	26.3	71.1	66.7	1.01
	133.32	1.83	7.45	24.6	68.0	64.4	0.99
	166.65	1.92	7.71	23.0	66.5	63.0	0.96
Transferrin	0 (Only micelle)	1.07	8.44	26.1	80.3	78.1	0.97
	33.33	1.47	7.67	28.0	78.9	75.6	1.00
	66.66	2.09	7.35	30.5	78.7	73.9	0.92
	99.99	2.57	7.14	30.0	77.5	71.6	0.98
	133.32	2.48	7.01	28.1	75.4	69.8	0.97
	166.65	2.27	7.56	18.4	48.9	45.8	1.06

Table S4. Secondary structural analysis of albumin, while interaction with SiQD-De/F127 nanoparticles.

Albumin (0.5 μM) + Micelle (μg/mL)	Helix-1 (%) Regular α-Helix	Helix-2 (%) Distorted α-Helix	Anti-3 (%) Right twisted β-strand	Others (%) irregular/loop
0.00	65.80	21.86	0.66	09.57
0.25	64.59	20.35	6.79	04.91
0.50	64.11	22.08	0	11.03
0.75	59.99	22.01	0	16.94
1.00	58.84	21.39	0	12.91
1.25	58.63	21.47	0	13.64
1.50	57.12	21.57	0	12.10
1.75	55.19	20.77	0	18.55
2.00	54.45	21.04	0	20.46
2.25	50.73	19.54	0	20.87

Table S5. Secondary structural analysis of fibrinogen, while interaction with SiQD-De/F127 nanoparticles.

Fibrinogen (0.2μM) + Micelle (μg/mL)	Helix-1 (%) Regular α-Helix	Helix-2 (%) Distorted α-Helix	Anti-1 (%)	Anti-2 (%)	Anti-3 (%)	Parallel	Turn	Others (%) irregular/loop
0.00	19.94	11.18	0	8.93	4.13	8.23	11.71	35.89
0.25	18.40	11.31	0	5.02	11.00	7.5	12.6	34.62
0.50	18.17	12.75	0	4.75	5.38	9.01	11.77	38.17
0.75	14.22	11.15	0	8.898	5.18	8.93	11.44	40.20
1.00	13.79	10.39	0	9.32	6.57	9.29	12.76	37.87
1.25	06.74	07.94	0	10.34	9.68	11.42	13.32	40.56
1.50	09.63	08.61	4.43	8.44	7.38	8.79	12.9	39.90
1.75	10.23	01.30	0	3.85	7.54	10.77	13.27	44.05
2.00	10.42	08.00	5.06	11.31	8.31	1.61	13.08	42.22
2.25	15.25	12.17	2.64	2.38	4.2	4.19	12.95	46.21

Table S6. Secondary structural analysis of transferrin, while interaction with SiQD-De/F127 nanoparticles.

Transferrin (1.0μM) + Micelle (μg/mL)	Helix-1 (%) Regular α-Helix	Helix-2 (%) Distorted α-Helix	Anti 3	Turn	Others (%) irregular/loop
0.00	26.14	15.10	0	6.45	52.30
0.25	24.76	11.39	1.51	7.69	54.64
0.50	20.93	9.85	3.35	10.08	55.79
0.75	18.97	9.88	0	12.12	59.03
1.00	10.81	5.54	21.10	10.58	50.18
1.25	14.77	7.83	24.21	10.35	42.83
1.50	14.91	5.55	18.82	10.60	49.97
1.75	19.04	6.67	8.95	11.65	53.69
2.00	20.84	10.82	0	11.30	57.04
2.25	25.06	10.12	6.54	8.56	49.71
